# People with limiting long-term conditions report poorer experiences and more problems with hospital care

**DOI:** 10.1186/1472-6963-14-33

**Published:** 2014-01-23

**Authors:** Paul Hewitson, Alex Skew, Chris Graham, Crispin Jenkinson, Angela Coulter

**Affiliations:** 1 Health Services Research Unit, Nuffield Department of Population Health, University of Oxford, Old Road Campus, Oxford OX3 7LF, Headington, England; 2 Picker Institute Europe, Buxton Way, 3 West Way, Oxford OX2 0JB, England

**Keywords:** Patient reported experience, Questionnaire development, Long term conditions, Questionnaire survey, Patient satisfaction

## Abstract

**Background:**

Long-term conditions have a significant impact on individuals, their families, and the health service. As people with these conditions represent a high proportion of hospital admissions, investigating their experiences of inpatient care has become an important area of investigation. We conducted a secondary analysis of the NHS adult inpatient survey for England to compare the hospital experiences of three groups of patients: those without long-term conditions, those with a single long-term condition, and those with multiple long-term conditions. We were particularly interested in the extent to which these patients received self-management support from hospital staff, so we developed a brief summary tool drawn from salient questions in the survey to aid the comparison.

**Methods:**

Analysis of data from the 2011 national adult inpatient survey (*n* = 65,134) to compare the experiences of three groups of patients: those with no limiting long-term conditions (No-LLTC), those with one limiting long-term condition (S-LLTC), and those with two or more limiting long-term conditions (M-LLTC). The main outcome measure was patients’ self-reports of their experience of inpatient care, including staff-patient interactions, information provision, involvement in decisions and support for self-care and overall ratings of care. A short form scale, the Oxford Patient Involvement and Experience scale (OxPIE) was developed from the adult inpatient survey and used to compare the groups using logistic regression.

**Results:**

There were significant differences between the No-LLTC group in comparison to both the S-LLTC and M-LLTC groups. Patients with limiting long-term conditions reported significantly worse hospital experiences than those without, as measured by OxPIE: S-LLTC odds ratio = 1.23, 95% CI 1.03-1.48; M-LLTC odds ratio = 1.64, 95% CI 1.19 – 2.26. Responses to a single global rating question were more positive but not strongly correlated with OxPIE.

**Conclusions:**

Patients with LLTCs were more critical of their inpatient care than those with no LLTCs. Those with more than one long-term condition reported worse experiences than those with a single limiting condition. Simple rating questions may not be sufficiently sensitive to reflect important aspects of patients’ experience.

## Background

Chronic diseases, physical, psychological, sensory or cognitive disabilities (long-term conditions (LTCs)) have a significant impact on individuals and their families and on health and social care services., LTCs are very common, especially among older people. An analysis of patient data from Scotland found that, in 2007, 42% of the population had at least one chronic condition and 23% had two or more [1]. The proportion with at least one LTC rose to 50% at age 50 and 80% at age 65. Similar proportions of people with multiple LTCs have been found in studies of US and Canadian populations [2,3]. People with LTCs account for a disproportionate share of hospital attendances, with LTCs accounting for 70% of hospital inpatient bed days in England in 2009 [4]. Those with long-term conditions that limit their activities (LLTCs) are the most intensive users of the most expensive services. There is evidence that strategies to engage, support and empower people with LTCs have an important role in improving health outcomes [5,6]. Surveys show that people with LTCs want to be involved in decisions about their care and they want access to information to help them make those decisions [4]. They also want their role in self-care to be acknowledged by staff and to be given effective support to help them self-manage their condition [7]. Most patients with LTCs in England report having some sort of care planning discussion with their GP, but only a small proportion experience proactive, systematic support in primary care [8,9]. This type of support is equally important during hospital episodes, since adequate preparation for returning home after discharge can be crucial to promote recovery and independent living. We have been unable to find any studies focusing on self-management support for hospital inpatients with LTCs. We therefore conducted a secondary analysis of data from the NHS national inpatient survey to examine the extent to which patients with and without LTCs receive self-management support during hospital stays.

Most published studies of patients’ experience have focused on the relationship between specific conditions (e.g. diabetes, cardiovascular disease) or multiple conditions (two or more LTCs), and health-related quality of life, medical services use or health care utilisation costs [10-12]. Most of these studies narrowly define LTCs as the presence or absence of a LTC, rather than whether or not the LTC(s) have an impact on peoples’ daily activities. The few studies which have investigated this issue in more depth underline the importance of incorporating self-reported burden associated with the LTC(s) to establish a more accurate understanding of peoples’ experiences and healthcare needs [13-15]. Since some LTCs (for example hypertension) may not affect the type of support people require in hospital, we chose to limit our analysis to those survey respondents who indicated that they had one or more LTCs that limited their activities.

The study had two main aims:

1. To develop an instrument, using psychometric methods, that would enable us to summarise patients’ reports of their experience, focusing in particular on the extent to which hospital staff informed and engaged them and provided them with self-management support;

2. To compare the hospital experiences of three groups of patients: those without limiting long-term conditions (No-LLTC), those with a single limiting long-term condition (S-LLTC), and those with multiple limiting long-term conditions (M-LLTC).

The national patient survey programme for England, currently run by the Care Quality Commission (CQC), has conducted inpatient surveys each year since 2004 [16]. This postal survey is sent to samples of 850 adult patients consecutively discharged from each hospital trust in the country during a specified period. The questionnaire, which asks respondents a number of questions about their most recent hospital stay, includes items on interactions with hospital staff, involvement in treatment and discharge decisions, information provision and support for carers. It also asks about the presence or absence of LTCs and whether these conditions limit their activities in any way. Although the results of the survey are reported publicly each year, detailed analyses of the experience of inpatients with and without LLTCs have not previously been published.

## Methods

### Data sources

We used data from the national inpatient survey that was conducted in 2011. For the purpose of this analysis we were interested in people who had a long term condition that limited their activities, excluding those with problems such as hypertension that might not have an obvious effect on what they are able to do. Two items in the inpatient questionnaire asked patients to indicate if they had a long-term condition (longstanding illness or physical condition, such as cancer, HIV, diabetes, chronic heart disease or epilepsy, hearing or vision problem, deafness or severe hearing impairment, blindness or partially sighted, mental health condition or learning disability) and, if so, whether this condition caused them difficulties in various aspects of their daily life (everyday activities, work, education or training, access to buildings, streets or vehicles, people’s attitudes to them, communicating, mixing with others or socializing, or any other activity). Respondents answering yes to both these questions were deemed to have a limiting long term condition (LLTC). We categorised respondents into one of three groups: no limiting long term condition (i.e. no condition that limited their activities, No-LLTC), a single limiting long term condition (S-LLTC), and multiple (two or more) limiting long term conditions (M-LLTC).

### Scale development

**Figure 1:**
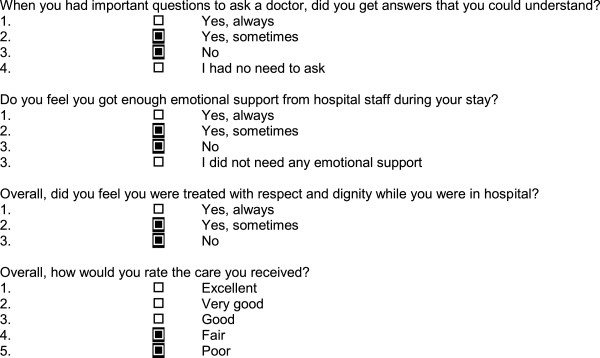
**Examples of questions showing derivation of problem scores.** Note: Black boxes indicate responses coded as a ‘problem’.

We transformed all thirty-one items in the questionnaire that specifically concerned patients’ experience of interpersonal care, including staff-patient interactions, information provision, involvement in decisions and support for self-care, into dichotomous variables. Other questions about waiting times, food and the environment were omitted from the analysis. We coded each of the items as a dichotomous ‘problem score’, indicating the presence or absence of a problem. A problem is defined as an aspect of healthcare that could, in the eyes of the patient, be improved upon to enhance their experience of care (Figure 1).

We used these items to develop a short summary measure that we named the Oxford Patient Involvement and Experience (OxPIE) scale. The criteria used to develop OxPIE were based on previously published methods for developing psychometrically reliable scales of inpatient experiences [17,18]. These criteria include:

1. items included in the short-form should be applicable to as many respondents as possible (i.e. very low proportion of missing responses to items)

2. the short-form created from the 31 pertinent items in the Picker adult inpatient questionnaire should ideally correlate with the longer ‘parent’ measure at 0.9 or above [19]

3. the level of internal consistency for the short-form (KR-20 for dichotomous variables) should be above 0.7 [20]

4. item total correlations, corrected for overlap, should exceed 0.3 for items in the short-form measure [21].

### Analysis

We carried out a bivariate analysis of categorical variables using the chi-squared test. Item analysis for the scale was based on an examination of the data for floor and ceiling effects, correlations between items, item discrimination and ordering of response categories. Internal consistency was evaluated with item-to-item and item-total correlations using the Kuder-Richardson (KR20) method [22]. We constructed a multivariable logistic regression model to examine associations between the OxPIE scale and key patient variables (LLTC status, age group, gender, length of stay, ethnicity, pain experienced during hospitalisation, having an operation or procedure during hospitalisation and route of admission). As older age is closely associated with LLTC status, interaction terms for age group and LLTC status were also included in the model. A backward-selection procedure, with a P value of less than 0.1 used for retention in the model, was used to identify important patient factors at the 0.05 level of statistical significance. All statistical analyses were performed using STATA (Version 10.1; StataCorp).

Picker Institute Europe has ethical approval to conduct the annual national Inpatient Survey from the National Research Ethics Service (NRES: 01/8/090) and NIGB Ethics and Confidentiality Committee (ECC) approval (under Section 251 approval from the Secretary of State) to process patient identifiable information derived from the Inpatient Survey (ECC 6-02 (FT1)/2012). This study used secondary analysis of publically available information (provided by Picker with the consent of CQC in the UK). At no time was any personal identifiable information made available to any of the researchers involved in this study.

## Results

### Respondents’ characteristics

The 2011 inpatient survey was mailed to 136,446 adult patients (aged 16 years and over) recently discharged from 161 acute and specialist NHS trusts in England. A total of 70,863 questionnaires were returned completed, a response rate of 51.9%. Of these, 65,134 respondents (91.9%) provided sufficient information about LLTC(s) to be included in the analysis. More women (53.2%) returned the questionnaire than men. Most respondents (92.0%) described their ethnicity as ‘white-British’, and nearly two-thirds (65.2%) were aged over 61 (Table 1). Overall, 55.8% of respondents reported no LLTCs, 29.0% had one and 15.2% had two or more. Approximately 32% of the No-LLTC group reported one or more LTCs but indicated that these did not limit their daily activities.

**Table 1:** Characteristics of respondents

		**No-LLTC *No.***	**Group *Pct.***	**S-LLTC *No.***	**Group *Pct.***	**M-LLTC *No.***	**Group *Pct.***	**Overall *No.***	**Total *Pct.***
**Gender**	*Female*	19,776	*54.4%*	9,919	*52.5%*	4,921	*49.7%*	34,616	*53.2%*
	*Male*	16,554	*45.6%*	8,982	*47.5%*	4,982	*50.3%*	30,518	*46.8%*
**Age group**	*16-29*	2,388	*6.6%*	758	*4.0%*	232	*2.4%*	3,378	*5.2%*
	*30-45*	4,113	*11.4%*	1,601	*8.5%*	551	*5.7%*	6,265	*9.7%*
	*46-60*	7,213	*20.0%*	3,989	*21.3%*	1,619	*16.7%*	12,821	*19.9%*
	*61-75*	12,725	*35.3%*	6,609	*35.2%*	2,976	*30.6%*	22,310	*34.6%*
	*75-90*	9,123	*25.3%*	5,392	*28.8%*	3,765	*38.7%*	18,280	*28.3%*
	*Over 90*	525	*1.45%*	406	*2.2%*	579	*6.0%*	1,510	*2.3%*
**Ethnicity**	*White-British*	33,254	*91.5%*	17,435	*92.2%*	9,267	*93.6%*	59,956	*92.0%*
	*Other*	3,076	*8.5%*	1,466	*7.8%*	636	*6.4%*	5,178	*8.0%*
**Admission to hospital**	*Emergency*	18,885	*54.0%*	11,149	*61.0%*	6,789	*71.1%*	36,823	*58.6%*
	*Planned*	16,084	*46.0%*	7,118	*38.9%*	2,756	*28.9%*	25,958	*41.4%*
**Operation or procedure**	*Yes*	24,728	*69.6%*	11,108	*60.2%*	5,141	*53.6%*	40,977	*64.4%*
	*No*	10,812	*30.4%*	7,346	*39.8%*	4,458	*46.4%*	22,616	*35.6%*
**Experienced pain**	*Yes*	22,468	*63.3%*	12,695	*69.0%*	6,741	*70.0%*	41,904	*70.0%*
	*No*	13,018	*36.7%*	5,705	*31.0%*	2,890	*30.0%*	21,613	*30.0%*
**Length of stay**	*1-3 days*	22,671	*62.4%*	9,462	*50.0%*	4,668	*47.1%*	36,801	*56.5%*
	*4+ days*	13,656	*37.6%*	9,435	*50.0%*	5,234	*52.9%*	28,325	*43.5%*
**Number of limiting long-standing conditions**	*0*	36,330	*100%*	-		-		36,330	*55.8%*
	*1*	-		18,901	*100%*	-		18,901	*29.0%*
	*2*	-		-		7,081	*71.4%*	7,081	*10.9%*
	*3*	-		-		2,293	*23.2%*	2,293	*3.5%*
	*4*	-		-		474	*4.8%*	474	*0.7%*
	*5*	-		-		46	*0.5%*	46	*0.0%*
	*6*	-		-		9	*0.1%*	9	*0.0%*
**Total**		36,330		18,901		9,903		65,134	*100%*

Patients in the M-LLTC group were more likely to have experienced an emergency admission than those in the other two groups: 71.1% of M-LLTC patients were admitted as emergencies as compared to 61.0% of the S-LLTC group and 54.0% of the No-LLTC group (*p* < 0.001). The majority of those in the No-LLTC group (69.6%) underwent operations or procedures, as compared to only half (50.0%) of those in the S-LLTC group and less than half (47.1%) of those in the M-LLTC group.

### OxPIE scale development

Twenty of the original 31 questions in the 2011 inpatient survey were omitted from the scale. These items were removed due to not being applicable to a large proportion of respondents (i.e. items not relevant to all respondents), or because their removal resulted in an increase in the reliability of the instrument. The item analysis for the OxPIE scale is shown in Table 2. The internal consistency of the OxPIE scale was good (KR-20 = 0.85) and the correlation between the eleven-item scale and the 31 item ‘parent’ measure was very high (r = 0.947; *p* < 0.001). Item-total correlations for each item in the scale were above the recommended level, indicating very good discrimination for each of the items.

**Table 2:** Items included in OxPIE scale including item-total correlation and percentage of missing values

***Item***	***Item content***	***Item total correlations***	***Missing values***
1.	Doctors’ answers to important questions not always clear	0.542	1.9%
2.	Nurses’ answers to important questions not always clear	0.568	1.6%
3.	Not easy to find someone to talk about worries and fears	0.590	2.1%
4.	Hospital staff sometimes gave conflicting information	0.484	2.0%
5.	Did not feel got enough emotional support from staff	0.604	1.9%
6.	Not always treated with respect and dignity	0.573	2.9%
7.	Not fully involved in decisions about care and treatment	0.563	2.3%
8.	Not fully involved in decisions about discharge	0.509	2.5%
9.	Not told who to contact if worried after discharge	0.347	4.4%
10.	Not told about danger signals to watch out for at home	0.512	3.7%
11.	Family/friends not given sufficient information	0.504	3.8%

Descriptive statistics for the OxPIE scale results for each of the three LLTC groups are shown in Table 3. Given the considerable skewness of the distribution of responses, the OxPIE scale was dichotomised using the median-split method [23,24]. The KR-20 for each of the LLTC groups was very similar to the KR-20 for the entire sample; No-LLTC = 0.83, S-LLTC = 0.85 and M-LLTC = 0.86.

**Table 3:** Descriptive statistics for OxPIE scale

	**Positive exp. (≤3)**	**Negative exp. (≥4)**	**Mean**	**SD**	**Range**	**25%**	**50%**	**75%**
All groups	54.7%	45.3%	3.76	3.23	0 to 11	1.00	3.00	6.00
No-LLTC	61.0%	39.0%	3.25	2.99	0 to 11	1.00	2.00	5.00
S-LLTC	49.0%	51.0%	4.19	3.33	0 to 11	1.00	4.00	7.00
M-LLTC	41.5%	58.5%	4.28	4.83	0 to 11	2.00	4.00	8.00

### OxPIE items

Detailed results for each of the OxPIE items are shown in Table 4. Across all age-groups, patients in the S-LLTC and M-LLTC groups consistently reported more problems with their experience of care than did those in the No-LLTC group. Patients in the M-LLTC group were more likely to report problems than those in the S-LLTC group for almost all of the items, and those in the younger age-groups reported more problems than those who were older.

**Table 4:** Problems with staff-patient interactions, information provision, involvement in decisions and self-care support by age and condition for items included in OxPIE

**Questionnaire items**	**Age group**	**No-LLTC %**	**95% CI**	**S-LLTC %**	**95% CI**	**M-LLTC %**	**95% CI**
Doctors’ answers to important	*16-29*	41.0	39.0-43.0	46.1	42.6-49.7	50.4	44.0-56.9
questions not always clear	*30-44*	34.9	33.4-36.4	39.7	37.3-42.1	50.5	46.2-54.7
	*45-59*	25.4	24.4-26.4	33.7	32.2-35.2	39.8	37.4-42.2
	*60-74*	18.3	17.6-18.9	29.0	27.9-30.1	35.9	34.2-37.6
	*75-89*	20.1	19.2-20.9	34.2	32.9-35.4	42.1	40.5-43.7
	*≥ 90*	30.4	26.3-34.4	34.9	30.1-39.6	51.4	47.3-55.6
	***Total***	***23.8***	***23.3-24.2***	***33.3***	***32.6-33.9***	***41.2***	***40.2-42.2***
Nurses’ answers to important	*16-29*	40.1	38.1-42.0	48.6	45.0-52.2	49.1	42.7-55.6
questions not always clear	*30-44*	35.1	33.6-36.5	39.9	37.5-42.3	45.7	41.5-49.9
	*45-59*	26.7	25.6-27.7	35.1	33.6-36.6	38.3	35.9-40.7
	*60-74*	20.7	20.0-21.4	31.2	30.1-32.4	37.2	35.5-40.7
	*75-89*	20.9	20.1-21.7	35.7	34.4-37.0	41.7	40.1-43.2
	*≥ 90*	28.7	24.8-32.7	42.7	37.8-47.6	48.3	44.2-52.4
	***Total***	***25.0***	***24.5-25.4***	***35.0***	***34.4-35.7***	***40.8***	***39.8-41.7***
Not easy to find someone to	*16-29*	45.4	43.3-47.4	48.3	44.8-51.9	59.4	53.0-65.8
talk about worries and fears	*30-44*	39.9	38.4-41.4	49.0	46.6-51.5	57.5	53.4-61.7
	*45-59*	32.7	31.6-33.8	41.9	40.4-43.5	47.9	45.4-50.4
	*60-74*	25.6	24.8-26.4	38.0	36.8-39.2	43.3	41.5-45.1
	*75-89*	26.8	25.9-27.7	42.1	40.8-43.5	48.0	46.4-49.6
	*≥ 90*	35.2	31.0-39.3	47.8	42.8-52.8	57.4	53.3-61.5
	***Total***	***30.4***	***30.0-30.9***	***41.6***	***40.9-42.3***	***48.1***	***47.1-49.1***
Hospital staff sometimes gave	*16-29*	48.0	46.0-50.1	60.3	56.8-63.8	69.7	63.8-75.7
conflicting information	*30-44*	40.3	38.8-41.8	50.4	48.0-52.9	59.2	55.1-63.3
	*45-59*	32.6	31.5-33.6	43.0	41.4-44.5	49.9	47.5-52.4
	*60-74*	24.5	23.7-25.2	36.4	35.2-37.5	43.1	41.3-44.9
	*75-89*	20.5	19.6-21.3	34.8	33.5-36.1	40.1	38.5-41.7
	*≥ 90*	26.1	22.2-30.0	36.2	31.4-41.0	44.0	39.9-48.2
	***Total***	***28.5***	***28.0-28.9***	***39.5***	***38.8-40.2***	***44.7***	***43.7-45.7***
Did not feel got enough	*16-29*	39.8	37.8-41.7	49.0	45.4-52.6	61.1	54.8-67.5
emotional support from staff	*30-44*	36.3	34.8-37.8	46.3	43.8-48.7	58.1	53.9-62.2
	*45-59*	26.7	25.7-27.8	38.5	37.0-40.0	44.8	42.4-47.3
	*60-74*	17.8	17.2-18.5	30.8	29.6-31.9	36.3	34.6-38.1
	*75-89*	17.0	16.2-17.8	31.9	30.6-33.2	37.5	35.9-39.1
	*≥ 90*	25.4	21.6-29.2	39.1	34.2-44.0	50.1	45.9-54.2
	***Total***	***23.1***	***22.7-23.5***	***35.1***	***34.4-35.7***	***41.0***	***40.0-42.0***
Not always treated with respect	*16-29*	34.1	29.5-33.3	39.7	26.2-43.2	47.4	40.9-53.9
and dignity	*30-44*	26.7	25.3-28.1	31.4	29.2-33.7	42.8	38.6-46.9
	*45-59*	19.0	18.1-19.9	25.5	24.2-26.9	32.6	30.3-34.9
	*60-74*	12.4	11.8-12.9	20.5	19.5-21.5	25.8	24.3-27.4
	*75-89*	10.9	10.3-11.6	21.2	20.1-22.3	26.9	25.4-28.3
	*≥ 90*	18.4	15.0-21.7	27.8	23.4-32.3	35.2	31.3-39.1
	***Total***	***16.3***	***15.9-16.7***	***23.7***	***23.1-24.3***	***29.5***	***28.6-30.4***
Not fully involved in decisions	*16-29*	51.1	49.1-53.2	57.6	54.1-61.2	66.7	60.5-72.8
About care and treatment	*30-44*	48.7	47.1-50.2	54.9	52.5-57.4	62.8	58.8-66.9
	*45-59*	43.3	42.1-44.4	49.2	47.6-50.7	54.3	51.9-56.8
	*60-74*	36.9	36.1-37.8	46.9	45.7-48.1	52.4	50.6-54.2
	*75-89*	43.4	42.4-44.5	55.1	53.7-56.4	61.3	59.8-62.9
	*≥ 90*	55.2	50.9-59.6	63.3	58.5-68.1	70.3	66.5-74.1
	***Total***	***42.4***	***41.9-42.9***	***51.3***	***50.6-52.0***	***58.4***	***57.4-59.3***
Not fully involved in decisions	*16-29*	48.0	45.9-50.0	49.6	46.0-53.2	52.4	45.9-58.8
about discharge	*30-44*	43.6	42.0-45.1	47.8	45.3-50.2	52.9	48.7-57.1
	*45-59*	38.6	37.5-39.7	43.1	41.6-44.6	46.7	44.3-49.2
	*60-74*	35.2	32.5-34.1	46.2	39.4-41.8	50.9	43.3-46.9
	*75-89*	41.4	34.2-36.2	47.1	44.8-47.5	55.7	49.2-52.5
	*≥ 90*	38.4	37.0-45.7	52.4	42.1-52.1	55.4	51.6-59.8
	***Total***	***37.1***	***36.6-37.6***	***43.9***	***43.2-44.6***	***49.0***	***48.0-50.0***
Not told who to contact if	*16-29*	33.1	31.2-35.0	32.5	29.2-35.9	40.6	34.2-47.0
Worried about condition or	*30-44*	28.1	26.7-29.5	31.4	29.1-33.7	36.8	32.7-40.9
treatment after discharge	*45-59*	23.5	22.5-24.5	25.7	24.3-27.0	30.6	28.3-32.8
	*60-74*	21.4	20.7-22.1	26.4	25.3-27.4	29.2	27.6-30.9
	*75-89*	31.8	30.8-32.8	36.8	35.5-38.1	41.6	40.0-43.2
	*≥ 90*	55.1	50.6-59.5	55.9	51.0-61.0	58.8	54.7-63.0
	***Total***	***26.5***	***26.1-27.0***	***30.5***	***29.9-31.2***	***37.0***	***36.0-37.9***
Not told about danger signals	*16-29*	52.0	50.0-54.0	52.1	48.5-55.7	57.8	51.3-64.2
to watch out for at home	*30-44*	47.8	46.2-49.3	49.5	47.0-51.9	58.9	54.7-63.0
	*45-59*	41.2	40.1-42.4	44.2	42.6-45.8	48.3	45.8-50.8
	*60-74*	36.7	35.9-37.6	44.4	43.2-45.6	48.2	46.4-50.0
	*75-89*	39.2	38.2-40.2	50.4	49.0-51.8	53.7	52.0-55.3
	*≥ 90*	44.7	40.2-49.2	51.2	46.1-56.4	58.5	54.3-62.6
	***Total***	***40.7***	***40.2-41.2***	***47.0***	***46.3-47.7***	***51.8***	***50.8-52.8***
Family/friends not given	*16-29*	46.7	44.7-48.7	48.1	44.5-51.7	47.8	41.2-54.3
sufficient information to help	*30-44*	39.1	37.6-40.6	46.0	43.5-48.5	52.3	48.1-56.5
recovery	*45-59*	34.6	33.5-35.7	40.4	38.8-41.9	42.9	40.1-45.4
	*60-74*	29.7	28.9-30.5	38.4	37.2-39.5	41.2	39.4-43.0
	*75-89*	28.6	27.6-29.5	40.5	39.2-41.8	44.4	42.7-46.0
	*≥ 90*	34.0	29.8-38.2	43.0	38.0-48.1	49.2	45.0-53.4
	***Total***	***32.7***	***32.2-33.2***	***40.6***	***39.9-41.3***	***44.2***	***43.2-45.2***

### Overall rating of care

In contrast to their responses to the detailed questions about their experiences, respondents gave much more positive answers to a global rating question: ‘Overall, how would you rate the care you received?’ (Table 5). Even so, both the M-LLTC (12.7%) and S-LLTC (9.5%) groups were more likely to say their care was only ‘fair’ or ‘poor’ (*p* = 0.001) than the No-LTC group (6.0%). As for the other items, the highest proportion of unfavourable ratings was seen among younger people in the M-LLTC group, a fifth of whom indicated problems with some aspects of their hospital experiences. Responses to this question were not highly correlated with the OxPIE scores (Pearson’s correlation coefficient of 0.463).

**Table 5:** Overall rating of hospital care by age and condition

**Questionnaire items**	**Age group**	**No-LLTC %**	**95% CI**	**S-LLTC %**	**95% CI**	**M-LLTC %**	**95% CI**
Overall, care received rated as	*16-29*	14.2	12.8-15.6	17.4	14.6-20.1	20.6	15.3-26.0
‘fair’ or ‘poor’	*30-44*	11.0	10.0-12.0	13.7	12.0-15.4	19.2	15.9-22.5
	*45-59*	6.8	6.3-7.4	10.5	9.5-11.4	14.3	12.6-16.0
	*60-74*	4.1	3.8-4.5	7.8	7.2-8.5	11.2	10.0-12.3
	*75-89*	3.5	3.1-3.9	8.4	7.6-9.1	11.2	10.2-12.2
	*≥ 90*	7.7	5.4-10.0	10.9	7.9-14.0	16.8	13.8-19.9
	***Total***	***6.0***	***5.8-6.3***	***9.5***	***9.1-9.9***	***12.7***	***12.1-13.4***

### Multivariate logistic regression

A multivariate logistic regression analysis was performed to determine the extent to which these results might be accounted for by differences in the composition of the three LLTC groups (Table 6).

**Table 6:** Results of the logistic regression analysis between key patient characteristics and the dichotomised OxPIE scale

**Factor**	**Comparison**	**OR**	**95% CI**	***p-*** **value**
*Main effects*				
Pain	No vs yes	1.61	1.55 to 1.67	<0.001
Ethnicity	W/Brit vs other	1.35	1.26 to 1.45	<0.001
Gender	Male vs female	1.33	1.29 to 1.38	<0.001
Length of stay	≤3 days vs ≥4 days	1.26	1.21 to 1.31	<0.001
Operation	No vs Yes	0.84	0.80 to 0.87	<0.001
Admission	Emergency vs plan.	0.60	0.57 to 0.62	<0.001
Age 30-44	16-29 vs 30-44	0.79	0.70 to 0.88	<0.001
Age 45-59	16-29 vs 45-59	0.59	0.53 to 0.65	<0.001
Age 60-74	16-29 vs 60-74	0.41	0.38 to 0.46	<0.001
Age 75-89	16-29 vs 75-89	0.43	0.39 to 0.48	<0.001
Aged over 90	16-29 vs 90+	0.63	0.49 to 0.79	<0.001
S-LLTC	No-LLTC vs SLLTC	1.23	1.03 to 1.48	0.024
M-LLTC	No-LLTC vs M-LLTC	1.64	1.19 to 2.26	0.003
*Interaction effects*				
S-LLTC × Age 30-44		1.02	0.82 to 1.27	0.849
S-LLTC × Age 45-59		1.13	0.93 to 1.38	0.227
S-LLTC × Age 60-74		1.34	1.10 to 1.62	0.003
S-LLTC × Age 75-89		1.49	1.22 to 1.82	<0.001
S-LLTC × Aged ≥90		1.17	0.81 to 1.70	0.404
M-LLTC × Age 30-44		1.37	0.81 to 1.71	0.107
M-LLTC × Age 45-59		0.94	0.67 to 1.33	0.736
M-LLTC × Age 60-74		1.26	0.90 to 1.75	0.180
M-LLTC × Age 75-89		1.49	1.07 to 2.08	0.019
M-LLTC × Aged ≥90		1.54	0.99 to 2.40	0.055

Note: Age group and LLTC effects currently included as main effects are the effects for the base age group category (Aged 16–29 years) and base LLTC category (No-LLTC). *P*-value for the overall interaction for age group and LLTC was 0.001.

In the total sample, women had worse OxPIE scores than men (OR = 1.33; 95% CI: 1.29 to 1.38); people in minority ethnic groups had worse scores than those who classified themselves as White British (OR = 1.35; 95% CI: 1.26 to 1.45); those who experienced pain during hospitalisation had worse scores than those who did not (OR = 1.61; 95% CI: 1.55 to 1.67); and those who stayed in hospital more than four days had worse scores than those with shorter lengths of stay (OR = 1.26; 95% CI: 1.21 to 1.31). In contrast, patients admitted for an operation or procedure had better OxPIE scores than those who did not (OR = 0.84; 95% CI: 0.80 to 0.87); those undergoing planned admissions had better scores than those admitted as emergencies (OR = 0.60; 95% CI: 0.57 to 0.62), and older people had better scores than those in the youngest age-group (16–29 years of age).

Both groups with limiting LTCs were significantly more likely to report worse experiences after accounting for these other factors: S-LLTC group (OR = 1.23; 95% CI: 1.03 to 1.48) and M-LLTC group (OR = 1.64; 95% CI: 1.19 to 2.26). Importantly, LLTC status was shown to mitigate the positive effect of age in respect of OxPIE scores. Whereas the main effect for all age categories was significantly associated with more positive scores on the OxPIE, this trend was reversed for the interaction effects of age group and LLTC status. Indeed, almost all of the odds ratios for LLTC status and age group were above 1, although only three (S-LLTC × Age 60–74 OR = 1.34; 95% CI 1.10 to 1.62, S-LLTC × Age 75–89 OR = 1.49; 95% CI 1.22 to 1.82, and M-LLTC × Age 75–89 OR = 1.49; 95% CI 1.07 to 2.08) were significantly associated with worse OxPIE scores. The significant interactions with poorer ratings of care for two S-LLTC age groups (60–74 and 75–90) and one M-LLTC age group (60–74), indicated that LLTC status had more influence on patients’ experiences than age group alone; LLTC status essentially reversing the positive effect of age on experience of care.

## Discussion

We believe this may be the first study to compare the experiences of people with and without LLTCs in a large dataset. The results indicate cause for concern because they suggest that people with LLTCs report worse hospital experiences than those without, but a number of caveats must be borne in mind when interpreting these findings. First, a response rate of 51.9% means the sample of respondents may be biased in some way. It is known that certain population groups are less likely to respond to postal surveys, including males, younger adults, the very old, people in poorer health, and those in socio-economically deprived groups [25]. Second, we relied on patients’ self-reports to determine the presence or absence of limiting LTCs. These are unlikely to be inaccurate but we had no means of verifying this. And third, although the analyses show a very strong relationship between the presence of limiting LTCs and individual likelihood of reporting problems in hospital experiences, we cannot necessarily infer from these cross-sectional survey data that the presence of LLTCs is a causal factor in people receiving poorer standards of care. It is possible that people with LLTCs have higher expectations of care, or that their assessments of care are influenced by differential treatment outcomes, or that people who are sicker adopt a more jaundiced view of their experiences. Finally, there is a potential ambiguity in the distinction between the S-LLTC and M-LLTC groups. Questions in the survey do not ask respondents to attribute limited activities to particular conditions: thus the M-LLTC group might be more precisely defined as including people who report more than one long-term condition, of which at least one limits their daily activities. The clear differences in results for the two S-LLTC and M-LLTC groups implies that this ambiguity is minor and that the distinction is meaningful.

Some of the patterns we noted have been seen in other similar surveys. For example, a recent inpatient survey in Scotland found that patients with poorer health status and those admitted as emergencies were less likely to report a positive experience of hospital care [26]. It has often been found that younger people are more critical of their care than those in older age groups, and there is also an observed tendency for patients from minority ethnic groups to report worse experiences than those from the majority group [27]. Recent research has suggested that these differences are not accounted for by differences in prior expectations of care [28].

A cross-sectional survey such as this can point to potential problems, but lacks explanatory power. We cannot know from these results why patients with LTCs experience worse care, or if they really do. Qualitative studies may offer some insight into what is going on. For example, a Swedish study that used focus groups to explore doctors’ attitudes to caring for elderly hospitalised patients suggested that the complexity of their conditions and the extra time required for effective communication gave rise to feelings of professional inadequacy and frustration, which may have affected the quality of their interactions with these patients [29]. A US study found that patients with multiple conditions gave marginally more critical reports on their interactions with doctors than did patients without LTCs, but the differences were not great [30]. Longer hospital stays, poor communication between staff working in different specialty departments, and inflexible hospital routines may lead to greater fragmentation and discontinuities in care for these patients [31].

It is worth noting that patients’ responses to a global rating question were more positive than their answers to the other questions examined here, and the correlation between responses to this rating question and the more detailed questions included in OxPIE was weak. This suggests that a single rating question may not adequately capture many important aspects of patients’ experience of hospital care. This question has since been removed from the survey and replaced in 2012 with a new ‘overall’ item with more Likert-style response options that may perform better. Nevertheless, it will be essential for healthcare staff and policy-makers to take account of the multifaceted nature of patients’ experience if they are to derive a better understanding of how to improve the delivery of the service. Simple rating or satisfaction questions may not adequately capture the totality of people’s experiences, in particular the relational, as opposed to transactional, factors that are so important to most patients.

## Conclusions

The fact that people with LTCs reported less satisfactory communications with staff, less involvement in decisions about their care, and less self-care support requires further investigation. Since the data came from a cross-sectional survey, we cannot know whether these findings are a true reflection of worse care for people with LTCs or a tendency for these people to be more critical as a consequence of their condition. External factors may have affected their assessments. Some of these patients may have been admitted to hospital as a result of ineffective management in primary and community care or inadequate social care support. Others may have experienced accidents, falls or acute medical problems that necessitated a distressing emergency admission. Nevertheless, the OxPIE scale revealed significant differences in reported experience that are of concern. It is worrying that patients with multiple conditions were even less likely than those with single conditions to report positive experiences. These findings add support to the view that many people with LTCs might be better off if they were cared for in their own homes [32,33]. They also point to the need to look for ways to optimize hospital care for this patient group, since admissions are sometimes unavoidable.

People with LTCs are heavier users of health and social care services, so providing proper support to help them return to independent living should be a priority, both for their individual well-being and also for improving the efficient allocation of resources. It is particularly important that hospital staff do all they can to help these patients and their carers to self-manage their conditions, yet our study suggests that this type of supportive care is not universally available in NHS hospitals.

## Abbreviations

LLTC: Limiting long-term condition, a long-term condition (also known as a ‘chronic condition’) which the patient self-reports as limiting their daily activities; No-LLTC: − No self-reported long-term conditions or long-term conditions which were not reported to limit daily activities; S-LLTC: Single self-reported limiting long-term; M-LLTC: Multiple self-reported limiting long-term conditions (also known as ‘multimorbidity’); CQC: Care quality commission; NHS: National Health Service.

## Competing interests

The authors declare they have no competing interests.

## Authors’ contributions

AC and CG were involved in the conception and design of the study. CG and AS assisted in accessing the data and contributing to the design of the analysis. PH designed the statistical and psychometric analyses and undertook all analyses. AC and PH wrote the initial draft of the manuscript. All authors were involved in revising subsequent drafts of the manuscript and contributing to the editing of the manuscript. All authors approve the final manuscript.

## Authors’ information

PH is a senior research officer at the HSRU involved in the development and evaluation of psychometric scales and statistical analysis of datasets. CJ is the Director of the HSRU and involved in a number of projects including QORU-related research. AC is a health policy analyst and researcher specialising in patient and public involvement in healthcare. CG is Director of Survey Research at the Picker Institute Europe. AS is a Research Associate at the Picker Institute Europe. Both CG and AS are involved in the evaluation of the NHS Adult Inpatient Survey.

## Pre-publication history

The pre-publication history for this paper can be accessed here:

http://www.biomedcentral.com/1472-6963/14/33/prepub
